# Diet and Microbiota–Gut–Brain Axis: A Novel Nutritional Therapy

**DOI:** 10.3390/nu18081223

**Published:** 2026-04-14

**Authors:** Lakshmi Markonda, Wendy A. Henderson

**Affiliations:** Department of Biobehavioral Health Sciences, University of Pennsylvania, 418 Curie Boulevard, Philadelphia, PA 19104, USA; lakkivsr@nursing.upenn.edu

Recent evidence shows that trillions of microorganisms in the gastrointestinal tract (GI tract) act as active regulators of various metabolic functions, including but not limited to neurologic function, mood, sleep quality, cognitive performance, and pain perception. Accurate assessment and microbiota modification through dietary and nutritional interventions are fundamental to nutrition and mental health research [1]. The profound influence of microbial composition and function on physiological processes has given rise to a paradigm shift in nutrition conceptualization. Hence, the bidirectional gut–brain axis has further evolved into the microbiota–gut–brain axis [2,3], a dynamic and increasingly consequential field of nutritional science. Despite decades of research on the gut–brain–microbiota axis, precision nutrition remains an active area of inquiry. Multiple interdependent mechanisms (microbial metabolites, neurotransmitter synthesis, intestinal barrier integrity, immune regulation, blood–brain barrier function) vary across individuals and are driven by genetics, epigenetics, or other external stressors such as diet restrictions. The multiple interdependent mechanisms complicate the identification of personalized therapeutic targets [4,5]. At the same time, mental health disorders, including depression, anxiety, and cognitive impairment, affect hundreds of millions of individuals globally [6].

While existing neuropharmacological measures are beneficial, they carry significant side effects and often do not address the underlying biological mechanisms driving neuropsychiatric pathology [7,8]. The individual variation in host genetics, baseline microbiota composition, dietary patterns, and genetic factors that affect nutrient absorption necessitates a more personalized precision nutrition approach to microbiota-targeted therapeutics [9,10]. The emergence of nutritional psychiatry targeting the gut microbiome with psychobiotic interventions offers a novel pathway forward [11]. Likewise, diet and nutrition-based interventions may simultaneously target multiple pathways implicated in the microbiota–gut–brain axis, potentially offering both efficacy and tolerability while addressing the root causes of dysbiosis [12,13]. Psychobiotics (probiotics and prebiotics) that confer mental health benefits through intestinal microbiota modulation represent a particularly promising therapeutic category, with preliminary evidence that suggests utility across depression, anxiety, cognitive impairment, and other psychiatric conditions [8,14].

In this Special Issue of *Nutrients*, there are exemplars of diverse research underway, internationally, to advance the evidence-informed clinical application of microbiota-targeted nutritional interventions. The issue comprises six peer-reviewed contributions that examine diverse methodological approaches, clinical populations, and mechanistic pathways within the microbiota–gut–brain axis. The six selected manuscripts highlight specific bacterial taxa and metabolic pathways linked to mental health outcomes and demonstrate the potential clinical benefits of psychobiotic interventions, especially in high-risk groups. Hence, the current Special Issue aims to advance nutritional therapeutics by demonstrating how dietary factors and changes in microbiota may influence brain health and psychiatric outcomes. Additional evidence is provided regarding microbiota-focused nutritional strategies that may be translated into practical interventions for psychiatric, neurological, and neurodegenerative disorders.

Costa and colleagues examined the oral–microbiota–visceral pain–brain pathway, identifying 259 operational taxonomic units and 471 genes associated with visceral hyperalgesia through transcriptomic profiling. The findings demonstrate that oral microbial profiles with reduced diversity are characteristic of participants with visceral hypersensitivity, with the bacterial families Lachnospiraceae, Prevotellaceae, and Veillonellaceae showing the highest association with induced visceral pain [15]. Gruenbaum and colleagues synthesized evidence linking the microbial regulation of glutamate (an excitatory neurotransmitter responsible for approximately 60% of all neurotransmitter activity) to brain health in post-neurological depression [2].

Kerstens and colleagues provided a comprehensive review on the influence of dietary patterns on oral and gut microbiota that promote or negate brain health. The data indicate that, while fermented foods enhance oral microbial diversity, fermentable carbohydrates benefiting gut microbes may simultaneously promote harmful oral bacteria [16]. Tao and colleagues examined the mental health indicators and gut microbiome while assessing the interactions of specific micronutrients (vitamins B1 and B2). The circulation of vitamins had significant associations with mental health outcomes including anxiety, stress, and sleep quality. The outcomes operated partially independent of microbiota composition shifts, suggesting direct vitamin-mediated effects on mood and cognition [14].

Climent and colleagues conducted a randomized trial in malnourished hemodialysis patients (a chronically ill population rarely studied in microbiota research), demonstrating that combined nutritional supplementation and probiotic administration reduced intestinal permeability biomarkers (lipopolysaccharides), decreased depression scores (Hospital Anxiety and Depression Scale), and increased quality of life scores (SF-12 mental health index). The aforementioned clinical applications demonstrate that psychobiotic interventions may confer measurable benefit in medically complex populations [17]. Mosquera and colleagues performed a systematic review of 51 randomized controlled trials involving 3353 patients, finding significant evidence for psychobiotic effectiveness in treating depression (present in 52.46% of reviewed studies), with more limited but suggestive evidence for cognitive impairment (10%), schizophrenia (10%), and bipolar disorder (6%) [8].

This Special Issue demonstrates how diet and microbiota modifications potentially influence brain health. Future research is warranted to prioritize standardization in the selection of the probiotic strain, dosage, treatment duration, and outcome assessment, with emphasis on human clinical efficacy rather than extrapolation from animal models [8,18]. Focused integration of microbiota composition, host genetics, dietary assessment, and baseline psychopathology to identify predictors of treatment response is recommended [9,10]. Although multi-omic profiling is complex, encompassing genomics, transcriptomics, proteomics, and metabolomics to map signaling pathways [15,17,19] is promising. The incorporation of oral microbiota profiling and oral health assessment as standard measures in microbiota–gut–brain axis studies is warranted. Instead of positioning conventional psychopharmacology and psychotherapy solely as an alternative, exploring the synergistic combination approaches through dietary modification and probiotic supplementation, alongside pragmatic trials in primary care, psychiatric, and primary prevention settings to evaluate population-level impact, is needed [8,14].

The six included studies represent the microbiota–gut–brain axis not merely as a biomedical curiosity but as a fundamental biological system through which food, resident microbes, and mental health are inextricably linked [2,20,21]. The current Special Issue demonstrates advancements in understanding microbiota-mediated mechanisms and in the use of targeted dietary and probiotic interventions, which have the potential to substantially improve mental health outcomes in future clinical practice. As the field matures and addresses current methodological gaps, working together across disciplines, institutions, and countries will provide researchers with opportunities to learn from one another and leverage resources to advance the field, ultimately improving mental health, preventing cognitive decline, and supporting healthy aging across the lifespan [8,14,16].
